# Breathing Pattern Monitoring by Using Remote Sensors

**DOI:** 10.3390/s22228854

**Published:** 2022-11-16

**Authors:** Janosch Kunczik, Kerstin Hubbermann, Lucas Mösch, Andreas Follmann, Michael Czaplik, Carina Barbosa Pereira

**Affiliations:** Department of Anesthesiology, Faculty of Medicine, RWTH Aachen University, 52074 Aachen, Germany

**Keywords:** contactless, classification, infrared thermography, RGB video, dataset, signal, extraction

## Abstract

The ability to continuously and unobtrusively monitor and classify breathing patterns can be very valuable for automated health assessments because respiration is tightly coupled to many physiological processes. Pathophysiological changes in these processes often manifest in altered breathing patterns and can thus be immediately detected. In order to develop a breathing pattern monitoring system, a study was conducted in which volunteer subjects were asked to breathe according to a predefined breathing protocol containing multiple breathing patterns while being recorded with color and thermal cameras. The recordings were used to develop and compare several respiratory signal extraction algorithms. An algorithm for the robust extraction of multiple respiratory features was developed and evaluated, capable of differentiating a wide range of respiratory patterns. These features were used to train a one vs. one multiclass support vector machine, which can distinguish between breathing patterns with an accuracy of 95.79 %. The recorded dataset was published to enable further improvement of contactless breathing pattern classification, especially for complex breathing patterns.

## 1. Introduction

Respiration provides the most crucial information to assess a patient for potentially life-threatening conditions. Various algorithms in emergency medicine demand verification and assurance of adequate breathing before other diagnostic or therapeutic steps are to be taken [1,2,3]. Even the decision to resuscitate an unconscious patient is only based on the absence of spontaneous breathing. Respiration is responsible for supplying the body with oxygen, essential for energy production and maintaining body functions. Furthermore, respiratory activity regulates the ${\mathrm{CO}}_{2}$ levels. Its removal during expiration directly influences the body’s pH levels. Hence, disturbed respiration is life-threatening, as all other body functions rely on it. As a result, many critical problems can be first observed in the respiratory activity before they manifest in other vital signs, such as heart rate, peripheral oxygen saturation, or blood pressure.

Furthermore, breathing patterns can be used as an indicator for various medical conditions. For example, traumatic injuries, cardiac, neurological, inflammatory, metabolic, and psychological conditions often lead to distinct changes in the breathing pattern. Therefore, an automatized monitoring of respiratory activity can give valuable information about the patient’s health state and could be used to prevent a broad spectrum of fatalities by allowing early detection of these conditions.

The current state of the art for breathing-pattern assessment utilizes respiratory belts strapped around a subject’s chest. While this approach is feasible for clinical research and sleep labs, it is unsuitable for long-term monitoring. Hence, contactless assessment of respiratory patterns would be desirable as it would allow monitoring a broad range of patients in normal wards, nursing homes, and even at home for various medical conditions.

### 1.1. State of the Art and Scope of this Work

The fact that contactless modalities can sense vitals has been shown by numerous publications. Most approaches in the literature utilize RF-based methods, such as radar, WiFi, or software-defined radios. Camera-based approaches using RGB cameras, Infrared Thermography (IRT), or Near Infrared (NIR) cameras are also widespread. Plentiful approaches have been published on extracting robust Respiratory Rates (RRs) from any of these modalities [4]. However, more features than RRs alone are required to classify a wide range of breathing patterns. The extraction of the required respiratory features and the classification of breathing patterns have yet to be presented to a satisfactory level.

Romano et al. [5] and Barbosa Pereira et al. [6] both performed a study in which multiple subjects were asked to breathe following an on-screen animation with different respiratory patterns. The experiments were recorded with RGB and IRT cameras, respectively. The data was then used to validate and compare multiple contactless signal extraction algorithms against a gold standard by comparing the RR extraction quality. However, neither a classification of the different breathing patterns was attempted, nor were additional respiratory features extracted.

Rehman et al. [7] showed that respiratory pattern classification based on software-defined radio measurements is possible and presented classifiers that were able to do so with good accuracy. However, as the description of the study protocol was kept very brief and the recorded dataset was not published, questions remain about the meaningfulness of these results. For example, excellent results could have been achieved by accidentally choosing unique RRs for each recorded breathing pattern, which would have reduced the classification problem’s complexity to only robust RR extraction.

So, the objective of this work was to build upon the idea of conducting a study in which volunteer subjects are asked to breathe according to a predefined signal containing multiple breathing patterns while recording their respiration with IRT and RGB cameras, as well as respiratory belts. Data augmentation was used to ensure that the resulting dataset covers the range of expected respiratory patterns and prevents overfitting. Subsequently, different algorithms were compared to determine the most suitable modality and extraction algorithm for contactless respiratory pattern classification. Furthermore, an algorithm for extracting multiple respiratory features was developed and evaluated. These features were used to train a classifier capable of distinguishing respiratory patterns. The full dataset was released alongside this publication to make all results verifiable, enabling the development of other classification approaches and allowing for a standardized comparison.

### 1.2. Respiratory Patterns

**Figure 1:**
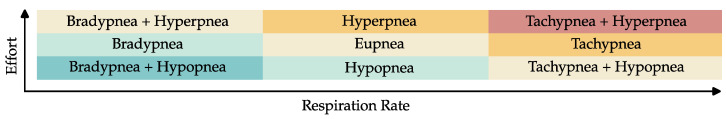
Qualitative respiration rate- and depth-based pattern differentiation. Hyperventilation and hypoventilation are marked by red and blue, respectively. The more saturated the color, the more pronounced the condition.

Descriptions of various breathing patterns are found primarily in medical textbooks (see, e.g., [8,9,10,11]). However, they often contradict each other. Hence, this section will discuss the relevant breathing patterns for this publication. Normal respiration is called eupnea and is defined by nearly sinusoidal thorax movements with RRs between 12 and 18 breaths/min[12]. Tachypnea is characterized by higher RRs than eupnea. It can be caused by either an increased oxygen demand (e.g., during exercise or fever), a reduced gas exchange capability (e.g., through asthma, pneumonia, pulmonary embolisms), or an oversupply of ${\mathrm{CO}}_{2}$ through, e.g., metabolic acidosis. The reasons for tachypnea can also be coped with an increased Respiratory Effort (RE) (deeper breaths), which is then called hyperpnea. Kussmaul breathing and hyperventilation are often used synonymously, which is not correct. Following the original publication from Kussmaul [13], deep, labored breaths with increased frequency define his discovered pattern, which can be seen in patients with severe metabolic acidosis. Hence, Kussmaul’s breathing is a combination of hyperpnea and tachypnea. Hyperventilation denotes a pathologically increased exchange caused by tachypnea, hyperpnea, or a combination of both. Figure 1 shows this interaction between the different breathing patterns. Hypoventilation is defined as a pathologically reduced gas exchange. Bradypnea and hypopnea, defined by lower RRs or REs, respectively, are common reasons for hypoventilation. However, in contrast to hyperventilation, it can also be caused by decreased pulmonary diffusion capability and manifest itself in tachy- or hyperpnea. Hence, there are exceptions to the color scheme in Figure 1.

**Figure 2:**
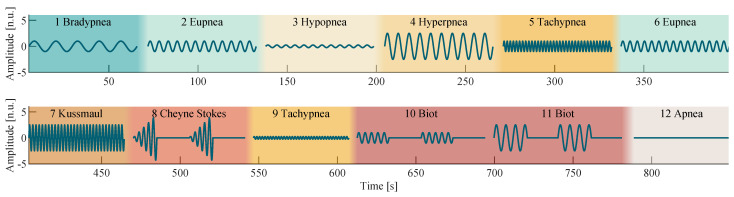
Visual representation of the breathing-pattern reference signal. A pause is placed between each breathing pattern to allow the subjects to breathe freely.

Breathing is controlled by the respiratory center, located in the brain stem as part of the autonomic nervous system. Because the respiratory center is near the narrow craniocervical junction, where the spinal cord leaves the cranial cavity, it can be disturbed by increased intracranial pressure, traumatic brain injuries and meningism (inflammation of the protective membranes of the spinal cord). Intoxication can also influence its functions. All the conditions mentioned above can reduce its activity and thus lead to bradypnea and hypopnea, which can even develop into respiratory arrest (apnea). Substantial impairments of the respiratory center manifest themselves as a pathological pattern called Biot’s breathing, characterized by periods of sufficient breathing, randomly interrupted with phases of apnea. An oxygen deficiency of the brain stem, for example, due to a stroke or heart attack, can yield a breathing pattern called Cheyne–Stokes, which is like Biot’s breathing defined by intermitted phases of apnea, but with increasing and decreasing REs. Figure 2 shows a visual representation of all discussed respiratory patterns.

## 2. Materials and Methods

### 2.1. Reference Signal Synthesis

Based on the research presented in the previous section, the breathing model

(1) $$s_{ref}\left(t\right)=\mathrm{RE}\;\cdot \;\sin\left(2\pi \;\cdot \;\frac{\mathrm{RR}}{60}\;\cdot \;t\right)$$

was utilized to synthesize a sequence of predefined breathing patterns as shown in Figure 2 by using the parameters specified in Table 1. A sinusoidal breathing model was chosen to synthesize the breathing patterns, as it reproduces the breathing movements of the thorax well.

Simple breathing patterns (with constant RR and RE) were chosen to have a length of 60 s. Two 20 s sections with respiratory activity were interleaved with two apnea sections of the same length to create 80 slong Biot’s breathing patterns. In the case of Cheyne–Stokes, the sections with respiratory activity were 15 s long and interleaved with apnea sections of length 20 s. The RE was linearly increased over time to approximate its characteristic alteration:

(2) $$RE\left(t\right)=\frac{\mathrm{RE}}{15}\;\cdot \;t.$$

The sequence of breathing patterns shown in Figure 2 was designed and tested to alternate in a way that a physiological gas exchange occurs on average, allowing subjects to breathe according to this sequence without risking hyperventilation or hypoxia. Between each breathing pattern, a pause of at least 5 s was added to the signal to allow the subjects to breathe freely. Because the shown signal was used as a reference during recording and evaluation, it is henceforth called the reference signal.

### 2.2. Data Collection

Training and validation data were collected through a voluntary subject study with 10 male and female subjects aged 24 to 45. During a 15-minute-long recording, all subjects were asked to sit comfortably in the provided chair and to breathe according to the reference signal. In advance, each subject was informed about the exact purpose and procedure of the study. Furthermore, it was expressly pointed out that the test protocol only needs to be followed at one’s discretion. All subjects were informed that they could interrupt the experiment at any time if, e.g., breathing patterns were causing discomfort. The study was conducted after notification of the Ethics Committee of the University Hospital RWTH Aachen University.

**Figure 3:**
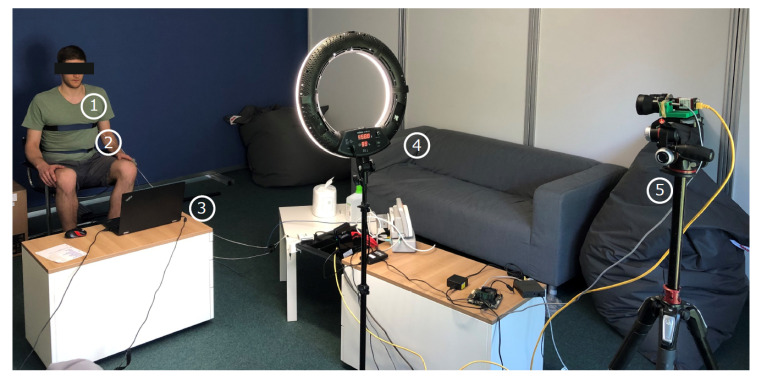
Experimental setup: A voluntary subject is sitting in a chair, wearing two respiratory belt sensors around the thorax (**1**) and abdomen (**2**). A computer (**3**) displays predefined breathing patterns and stores the video frames of an RGB and thermal camera (**5**). Illumination is ensured by using a ring light (**4**).

**Figure 4:**
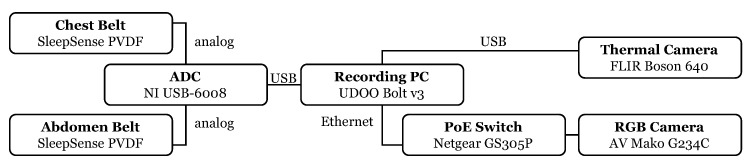
Diagram of the measurement setup.

Data were collected as shown in Figure 3 by using three different sensor modalities. Two respiratory belt sensors, placed around the thorax (1) and abdomen (2) of the subject, served as gold-standard signal sources, which recorded chest and abdominal breathing motions. An RGB and a thermal camera (5) were used to record the upper body of the sitting subject. A ring light (4) between the subject and the cameras ensured proper illumination. The reference signal was displayed on a computer (3) in front of the subject as an animation. Figure 4 shows a diagram of the measurement setup, including all used devices and interfaces. Detailed information about the devices and their settings can be found in Table A1 in Appendix A. MATLAB [14] was the software used for data collection, algorithm development and evaluation.

The readings from chest and abdomen belt sensors were oversampled with 5 kHz, digitally low-pass filtered by using a moving mean filter, and then downsampled to $f_{s}=10\mathrm{Hz}$. This sampling rate allows for the reconstruction of respiratory signals with up to three harmonics for RRs up to 100 Breaths/min. The extracted signals, from here on referred to as chest belt and abdomen belt, were used as the gold standard comparison for the contactless extracted signals.

**Figure 5:**
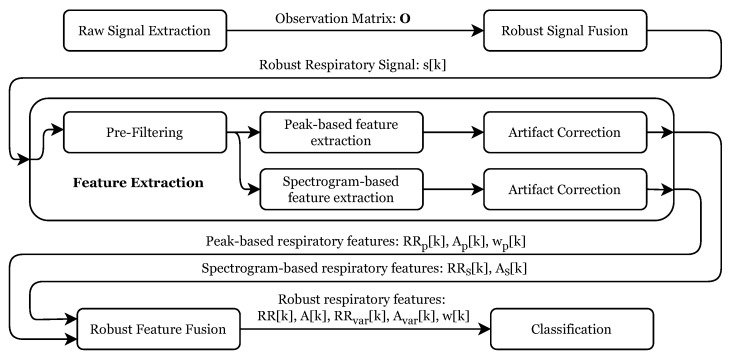
Overview of the complete signal processing algorithm from the raw signal extraction to the breathing pattern classification.

The performed signal processing is described in the following. Figure 5 gives an overview of the individual processing steps. After the raw signal extraction, these signals were merged into a robust respiratory signal, which was used to extract several respiratory features robustly. These features were then used as input for the final signal classification stage. The following subsections describe every step in detail.

### 2.3. Raw RGB Signal Extraction

**Figure 6:**
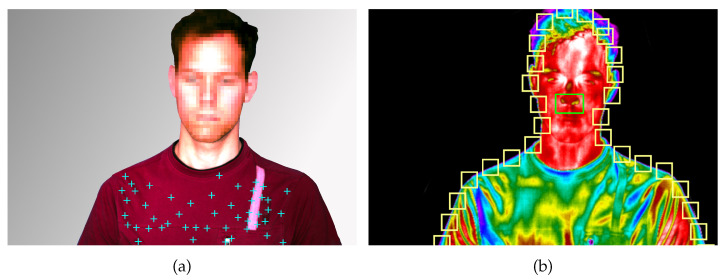
Points and regions of interest for respiratory signal extraction for color and thermal videos: (**a**) Spatially equally distributed minimum eigenvalue feature points on subject’s chest; (**b**) Region of interest for thermal videos at border between subject and background (yellow) and around the subject’s nose.

Respiratory signal extraction from RGB footage was achieved by tracking the subjects’ chest motion. For this purpose, 50 feature points were selected from this region (see Figure 6a), by using the minimum eigenvalue algorithm [15]. These points were used to set up and run a Kanade-Lucas-Tomasi (KLT) tracker [16,17], which tracked their positions in Cartesian coordinates over the full length of the video. To transform the *n* individual, two-dimensional trajectories $T_{i}\in R^{mx2},i\in \left[1,n\right]$ with length *m* into one-dimensional signals, a Singular value decomposition (SVD) was utilized:

(3) $$T_{i}=U_{i}\Sigma_{i}V_{i}^{*}.$$

The SVD is a generalized form of the eigenvalue decomposition for non-square matrices and uses two change-of-basis matrices $U_{i}\in R^{2x2}$, $V_{i}^{*}\in R^{mxm}$ to rotate the signal’s vector space, such that the semi-major and semi-minor axes of an ellipse, fitted to the signal distribution align with the coordinates’ system’s base vectors. In this case, $U_{i}$ and $V_{i}^{*}$ represent base change matrices for the spatial and temporal domains, respectively. Multiplying the two-dimensional trajectories with the first column vector of $U_{i}$, called ${\vec{u}}_{i,1}$:

(4) $${\vec{o}}_{i}=T\;\cdot \;{\vec{u}}_{i,1},$$

reduced their spatial dimensionality to only the primary direction of motion. The results were collected in the observation matrix $O\in R^{mxn}$

(5) $$O=\left[\begin{array}{ccccc} {\vec{o}}_{1} & ... & {\vec{o}}_{i} & ... & {\vec{o}}_{n} \end{array}\right],$$

which is composed by *n* trajectories ${\vec{o}}_{i}\in R^{m},i\in \left[1,n\right]$ with length m. These signals are referred to as chest RGB.

### 2.4. Raw Thermal Signal Extraction

The respiratory signal extraction from thermal videos was performed by using three independent approaches:

- observation of temperature changes below the nostrils (referred to as nose IRT),
- tracking the subject’s upper body movement in contrast to the background (referred to as border RoI) and
- tracking of feature point on the subject’s chest, analog to the RGB-based approach in Section 2.3 (referred to as chest IRT).

The first approach was investigated in multiple publications [18,19,20] and is based on the fact that inspiration cools and expiration warms the nostrils. Hence, their temperature change can be used as a respiratory signal. A Region of Interest (RoI) around the subject’s nose was selected (see Figure 6b) and tracked for this purpose using minimum eigenvalue [15] feature points in the subject’s face and a KLT tracker [16,17]. The RoI’s mean temperature was extracted in every frame and was directly used as respiratory signal s[k].

The second approach utilizes the naturally high contrast between the subject and the background. For this purpose, the first recorded thermal frame was automatically segmented into the foreground and background by using Otsu’s method [21]. Subsequently, all regions in the foreground mask were ordered by their area. The region with the largest area was treated as a mask for the test subject. To extract respiratory signals, n=30 RoIs with a size of 25 × 25 pixels were initialized along the border of the test subject and the background (see Figure 6b). Their mean value was computed for every video frame and again saved in an observation matrix O. Finally, the two-norm of the difference of all consecutive frames was computed

(6) $$e_{m,k}=\left|\right|O_{k}-O_{k-1}{\left|\right|}^{2}$$

to detect non-respiration-induced movements of the test subject. If the motion error $e_{m,k}$ grew larger than 5, the frame was marked as unusable and discarded. Two seconds after the latest marked frame, all RoIs were re-initialized and the signal extraction was continued.

### 2.5. Robust Respiratory Signal Fusion

Not all signals in the observation matrices O are mainly influenced by the subject’s respiratory activity but may also contain motion artifacts or only consist of noise. These signals cannot be used for robust respiratory signal extraction and thus should be discarded. The correlation matrix of O was computed to decide which signals are suitable. It was assumed that respiratory activity yields a high correlation between all signals in which it can be observed. Thus, the mean correlation between a signal and all other observed signals was calculated:

(7) $$\rho_{\mu ,i}=\sum_{j=1}^{n}\frac{\rho_{j,i}}{n},i\in \left[1,n\right].$$

In this equation, $\rho_{j,i}$ represents a cell value of the correlation matrix. Only the five signals with the most significant mean correlation coefficients were kept in a reduced observation matrix $O_{max}$, which was then used as input for a moving window Principal component analysis (PCA) with a window length $w_{l}$ of 30 s:

(8) $$C_{k}=\left[\begin{array}{ccc} {\vec{c}}_{1}, & ..., & {\vec{c}}_{t} \end{array}\right]=PCA\left(W_{k}\;\cdot \;O_{max}\right).$$

The window matrix $W_{k}=1_{\left\{1,...,k-1,k+w_{l},..,m\right\}}\in R^{w_{l},n}$ is a submatrix of an m-dimensional identity matrix, which only selects the observation points $\left[k,k+w_{l}-1\right]$ and thus only provides a portion of the observation matrix to the PCA algorithm.

A PCA is an orthogonal, linear transformation that transforms the data into a new coordinate system, in which the first axis corresponds to the direction of the largest signal variance. Because the variance of a signal is independent of its sign, the transformation-coefficients C may change their sign for every move of the window, which causes discontinuities in the result. Therefore, the signs of the current coefficient matrix $C_{k}$ were adjusted to be identical to its predecessor, using the direction matrix $D_{k}$:

(9) $$D_{k}=sgn\left(C_{k}\circ 1\right)\;\cdot \;sgn\left(C_{k-1}\circ 1\right),$$

where ∘ is the Hadamard product. Finally, the robust respiratory signal s[k] at time k was computed by multiplying the observation vector ${\vec{o}}_{k}$ with the direction matrix D[k] and the first column vector ${\vec{c}}_{1,k}$ of the coefficient matrix C[k], which represents the signal’s principal component:

(10) $$s\left[k\right]={\vec{o}}_{k}\;\cdot \;D_{k}\;\cdot \;{\vec{c}}_{1,k}.$$

### 2.6. Dataset Creation and Augmentation

After the respiratory signal extraction from all modalities and approaches mentioned above was completed, the sequences were manually labeled by comparing them with the reference signal. Because the signal amplitudes varied noticeably for different modalities and subjects, all signals were normalized. For this purpose, the signal amplitudes of eupnea sections were calculated by finding the maximum amplitude in their frequency spectrum, using a Fast Fourier Transform (FFT). These amplitudes were defined to have the value 1 normalized unit (n.u.):

(11) $$s_{norm}\left[k\right]=\frac{1}{max(|F\left({\vec{s}}_{eup}\right)|)}\;\cdot \;s\left[k\right].$$

It was noted that all signals exhibited a slight systematic error in their sampling rate. Therefore, all signals of a modality were resampled to match the desired sampling rate and were then aligned with each other by finding the most likely delay $d_{i,j}$ between every signal pair, using their cross-correlation. By using a least squares optimization problem of the form

(12) $$\left[\begin{array}{c} d_{1,2}\\ \vdots \\ d_{i,j}\\ \vdots \\ d_{n-1,n} \end{array}\right]=\left[\begin{array}{ccccccc} 1 & -1 & \; & \cdots & \; & & 0\\ \; & \; & & \vdots & \; & & \\ 0 & \cdots & 1 & \cdots & -1 & \cdots & 0\\ \; & \; & & \vdots & \; & & \\ 0 & \; & & \cdots & \; & -1 & 1 \end{array}\right]\left[\begin{array}{c} d_{1}\\ \vdots \\ d_{i}\\ \vdots \\ d_{n} \end{array}\right],$$

these distances were converted into the specific signal offsets $d_{i}$. Some signals exhibited a larger error in their sampling rate and were discarded in the first resampling and alignment step. A grid search over all possible resampling factors was performed for those signals. The factors that yielded the highest cross-correlation value and their corresponding lag were chosen to resample and delay these signals accordingly. Next, the mean signals of all recordings per modality were computed to align the different modalities with each other. As before, using cross-correlation analysis, the delays $d_{i,j}$ between each signal pair were computed and transformed into the signal offsets $d_{i}$ by solving Equation (12). These offsets were then added to all signals of the specific modality. The dataset was then stored in the MIT Waveform Database (WFDB) format.

The original data was altered by combining arbitrary signals $s_{i}\left[k\right]$ and $s_{j}\left[k\right]$ of different subjects, with each other using random percentages p∈[0,1] to augment the dataset:

(13) $$s_{\mathrm{gen},l}\left[k\right]=p\;\cdot \;s_{i}\left[k\right]+\left(1-p\right)\;\cdot \;s_{j}\left[k\right].$$

**Figure 7:**
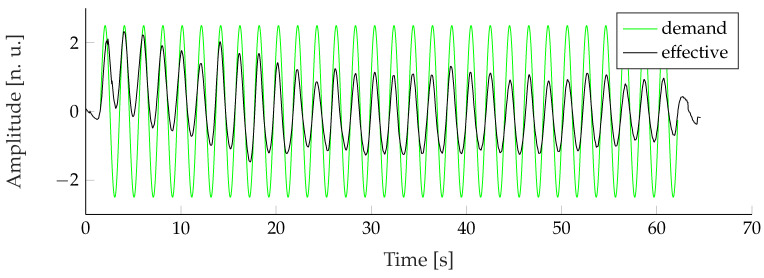
The recorded Kussmaul patterns were excluded from the dataset because of a big difference between demanded respiratory activity and the mean effective respiratory response of all subjects.

Afterward, the signals $s_{\mathrm{gen},l}\left[k\right]$ were split into the individual recorded breathing patterns. For each breathing pattern, the signal was either stretched or compressed to randomly vary the RR within its specified target range (see Table Section 2.1). This approach increased the original dataset size to 1000 samples for each pattern, modality, and extraction approach, allowing the utilization of any machine learning classifier. Furthermore, the RRs of the recorded signals were randomly distributed to prevent classifiers from simply distinguishing several patterns through their distinct frequency. Because the required RE of the recorded Kussmaul breathing pattern could not be replicated by any subject (see Figure 7), those sections were discarded and replaced with hyperpnea patterns that were shifted into the higher Kussmaul RR range.

### 2.7. Feature Extraction

In a pre-processing step, the respiratory signals s[k] were smoothed by filtering with a Savitzky–Golay filter [22] with the parameters shown in Table A2 in Appendix B. Then, by using MATLAB’s finpeaks algorithm, all peak locations $T_{p}\left[k\right]$, their amplitudes $a{\left[k\right]}_{p,\mathrm{nu}}=0.5\;\cdot \;p\left[k\right]$ (where p[k] is the peak prominence) and half-widths $w{\left[k\right]}_{p,\mathrm{nu}}$, which fulfilled the criteria given in Table A3 in Appendix B were extracted from the smoothed signal.

The RR was computed through the inverse of the continued differentiation between two consecutive peak locations $T_{p,k}$ and $T_{p,k-1}$:

(14) $$RR_{p,\mathrm{nu}}\left[k\right]=\frac{60}{T_{p,k}-T_{p,k-1}}.$$

$RR_{p,\mathrm{nu}}\left[k\right]$, $A_{p,\mathrm{nu}}\left[k\right]$ and $w_{p,\mathrm{nu}}\left[k\right]$ were nonuniformly sampled with the occurrence of the inspiratory peaks. Hence, they were resampled into $RR_{p}\left[k\right]$, $A_{p}\left[k\right]$ and $w_{p}\left[k\right]$ to match the length and sampling rate of s[k], by using a next-neighbor interpolation, which holds the last detected value until the next value.

The RR and its amplitude were also extracted from the signal’s spectrum by using Continous Wavelet Transform (CWT) ridges to increase the robustness of the feature extraction. Ridge points represent the local maxima of a spectrogram and indicate a signal’s instantaneous frequencies and amplitudes within the resolution of the used transform [23]. Hence, the RR and the respiratory amplitude were computed from continuous wavelet spectrogram S[f,k] of s[k] by finding the frequency f∀k, which maximizes the value of *S*:

(15) $$\begin{array}{cc} RR_{S}\left[k\right] & =60\;\cdot \;\underset{f}{arg max}\left(S\left[f,k\right]\right),\\ A_{S}\left[k\right] & =\max_{f}\left(S\left[f,k\right]\right). \end{array}$$

**Figure 8:**
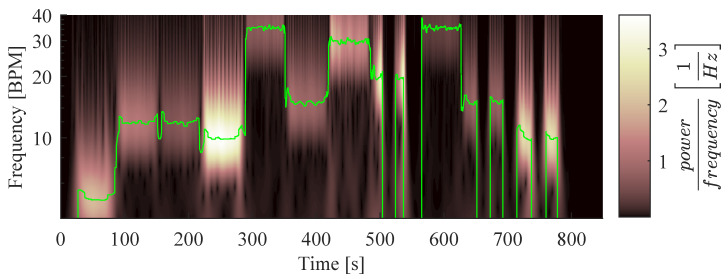
Continuous wavelet spectrum of the mean RGB signal, including the extracted respiratory rate.

The parameters used to compute S[f,k] can be found in Table A4 in Appendix B. For example, a CWT spectrogram with its ridge signal is shown in Figure 8.

### 2.8. Artifact Correction

Artifact correction was done on both feature tuples $F_{p}\left[k\right]=\left(RR_{p}\left[k\right],a_{p}\left[k\right],w_{p}\left[k\right]\right)$ and $F_{s}\left[k\right]=\left(RR_{S}\left[k\right],a_{S}\left[k\right]\right)$ individually. For ease of notation, the following equations are thus derived from a generic feature set $F_{i}\left[k\right]\;i\in \;\left[p,S\right]$.

Signal segments that were too discontinuous, showed noisy behavior below a certain respiratory amplitude, had a too low RR, or contained too few valid values, were invalidated. All steps greater than 5 Breaths/min were marked to remove segments with too many discontinuities in RR. The associated section was discarded if two discontinuities occurred within a 10 s time frame.

Signals with amplitudes below 0.05 n.u. were marked as unreliable. In order to nevertheless include the associated segments, three conditions had to be met: the segment $F_{i,j}\;\subset \;F_{i}$ had to be longer than five times the period length of its median RR, the median RR had to be greater than 5 breaths/min and the onto the median RR normalized difference between the smallest and largest RR had to be smaller than 0.45 n.u.:

(16) $$\begin{array}{cc} RR_{\mathrm{med}} & ={\tilde{RR}}_{i,j}\left[k\right],\\ \underset{\mathrm{in}\;[s]}{\mathrm{length}}\left(F_{i,j}\left[k\right]\right) & \geq \frac{5\;\cdot \;60}{RR_{\mathrm{med}}},\\ RR_{\mathrm{med}} & >5\;\mathrm{Breaths}/\min,\\ \frac{\max(RR_{i,j}\left[k\right])-\min\left(RR_{i,j}\left[k\right]\right)}{RR_{\mathrm{med}}} & <0.45\;n.u.. \end{array}$$

Lastly, all segments shorter than 10 s were also invalidated. All invalid segments shorter than 10 s were subsequently filled with a moving median interpolation of the last five valid values. Segments not satisfying this condition were set to zero.

### 2.9. Robust Feature Fusion

**Figure 9:**
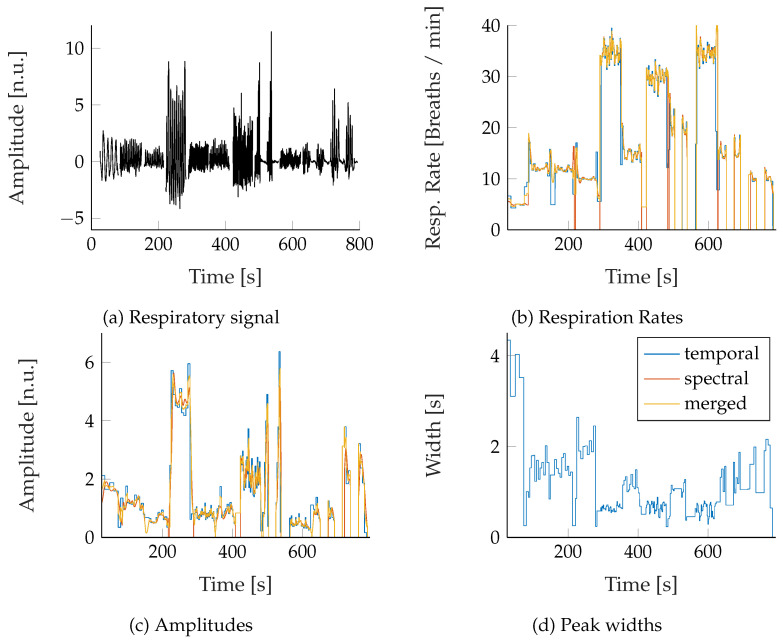
Time progression of the extracted features of an exemplary signal (RGB video of subject 6). All spectral, temporal and robustly merged features are displayed.

Figure 9 shows the peak- and CWT-ridge-based feature sets next to its underlying respiratory signal (Figure 9a). Though both sets were artifact corrected, it can be seen that they still exhibit some artifacts, independently from each other (For example, between 100–300 s in Figure 9b). Generally, it has been observed that the temporally extracted feature set was more reliable during highly transient conditions, like during Biot’s breathing and that the CWT-ridge method performed better in low- amplitude scenarios, like during tachy- and hypopnea.

Hence, the results of both approaches were merged into a robust feature set. For this purpose, all signal jumps greater than 5 breaths/min in the two respective RR were marked and used as segment borders. For each segment, the signals were merged with the following rules: If the difference between the median RR of both extraction approaches was smaller 3 breaths/min, the mean of both signals was used. Otherwise, the segment that had no signal jump in RR for at least 10 s and whose mean RR was higher compared to the other was used. If no valid section was present, the merged signal was set to an invalid value and later on filled by using a moving median interpolation over the last five valid values and a maximum signal gap of 5 s. Values that were still invalid afterward were set to zero. From the robust feature set, moving signal variances $RR_{\mathrm{var}}$ and $A_{\mathrm{var}}$ were computed with a time window of 30 s as a feature of change in the RR and RE.

### 2.10. Classification

A one vs. one multiclass Support Vector Machine (SVM) classifier was utilized for breathing pattern classification. The inputs to the classifier were chosen to be the features with $RR_{\mathrm{med}}$ and $A_{\mathrm{med}}$$RR_{\mathrm{var},\;\mathrm{med}}$ and $A_{\mathrm{var},\;\mathrm{med}}$ computed by calculating the median of the respective features over complete breathing pattern sections. SVMs were first introduced by Wapnik et al. [24] and are binary classificators that optimize a linear border between two labeled classes, such that the distance of their data points to the border is maximized. To allow the classification of multiple classes, in the one vs. one approach proposed by Hastie et al. [25], a classifier for every combination of two classes is trained. The classifier was trained and validated by using 10-fold cross-validation. Here, the dataset was randomly split into 10 subsets, from which, in every step, nine were used for training and one was used as validation data.

## 3. Results

### 3.1. Signal Extraction

**Figure 10:**
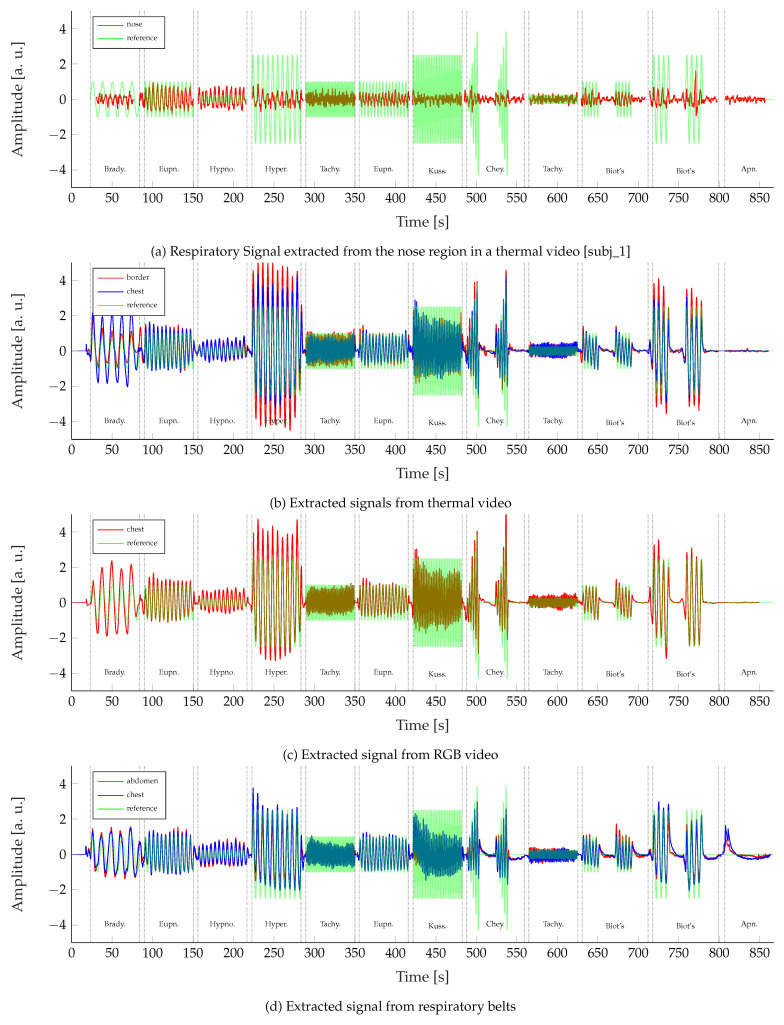
Temporal comparison of the mean signals of all modalities against the reference signal.

Figure 10 visually compares the extracted respiratory signals, grouped by their origin modality, against the reference signal, to which all subjects adjusted their breathing during the recordings. The results of the nose IRT signal extraction approach are shown on a single subject’s recording in Figure 10a. It can be seen that the signal oscillates with the reference, but no significant amplitude variations are visible. For this reason, the extraction approach was not further examined, as detecting amplitude differences between the breathing patterns is crucial for their correct classification.

Figure 10b displays the remaining respiratory signal extraction approaches for IRT videos. The displayed signals represent the mean of all extracted signals over all recorded subjects. An excellent agreement between both approaches and the reference signal can be observed regarding the frequency. Visible changes in amplitude during the different sections correlate well with the reference. However, it can be seen that the signal amplitudes are visibly larger for the chest IRT approach during bradypnea and the border RoI approach during hyperpnea. As the respiratory signals from RGB videos in Figure 10c were extracted in the same way as the chest IRT signals, both are very similar to each other. Only during phases of apnea does the IRT-based signal seem less noisy. On the other hand, the border-based IRT signal shows the most noise during those phases. Visually, the gold standard signals extracted from the respiratory chest and abdomen belts in Figure 10d have the best accordance with the reference signal. Here, the correlations between frequencies and amplitudes are excellent. Only during phases of apnea, the belt-based signals performed worse due to baseline wander.

### 3.2. Feature Extraction

Figure 9 in Section 2.8 shows the high temporal resolution of the extracted respiratory features. It can be noted that the fusion of the two redundantly extracted feature sets reduces the artifacts in the merged feature set. However, for signals with low REs, the CWT-ridge-based approach performed better than the peak detection approach, as single missed peaks significantly altered the feature extraction result. Furthermore, the accordance between the independently extracted feature signals can be used to indicate the overall extraction quality.

Table 2 shows the Root Mean Square Error (RMSE) for the different respiratory breathing patterns compared to the reference signal. All test subjects were asked to breathe according to this signal. Therefore, the RMSE values originate not only from the extraction algorithms’ performance alone but are superimposed with the errors the subjects made while breathing according to the reference signal. However, because these errors influence all extraction approaches equally, their comparison still unveils their relative performance against each other. Regarding RMSE, the chest belt shows the best performance. Nevertheless, the previously observed inferior performance during a respiratory arrest is also reflected in large error values in Biot’s respiration and apnea. The contactless modalities perform comparably well and show only slightly higher errors than the chest belt. However, due to the extremely high errors during phases of apnea for the chest belt signals, the mean RMSE of the contactless extracted signals outperforms the gold standard. The abdominally placed respiratory belt has the worst performance.

**Figure 11:**
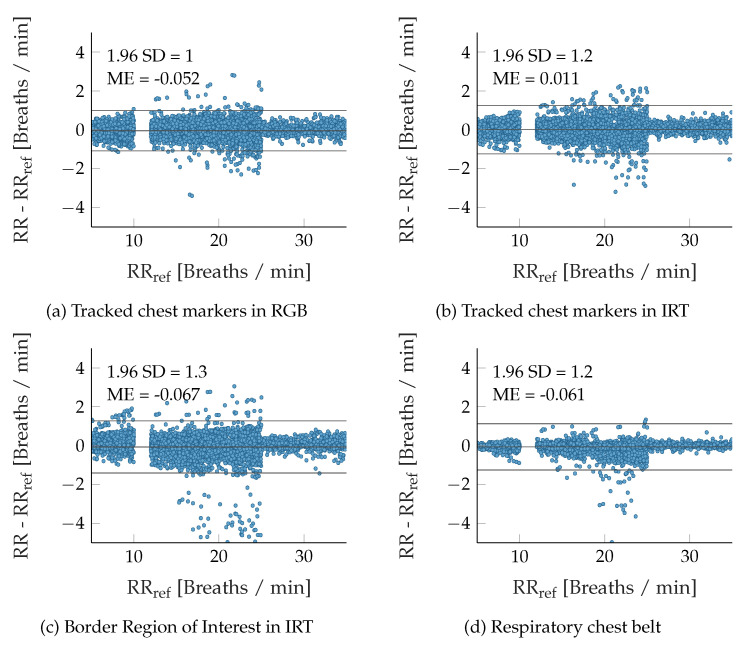
Bland–Altman diagrams for extracted respiratory rates.

Figure 11 compares the RR extraction results from contactless modalities and the chest belt signal (the gold standard) against the reference signal by using Bland–Altman plots. The latter signal served as ground truth. Hence, ${\mathrm{RR}}_{Ref}$ was used for the abscissa instead of the mean of RR and ${\mathrm{RR}}_{Ref}$, which would typically be expected from a Bland–Altman plot. In addition, the errors during the apnea phases were excluded. As in Table 2, it can also be seen that the median errors of the chest belt signals are significantly smaller. However, between 18 and 25 breaths/min the errors are considerably larger than the rest of the RR range. They show a bias toward estimating RRs to low, so that the standard deviations (SD) of all modalities and algorithms are very similar in the end. On average, all modalities show a similar, slightly negative mean error (ME), which means that RRs are estimated slightly too low. Only the RR extraction errors from chest IRT signals show a significantly lower, slightly positive bias.

Table 3 counts the number of outliers, which are greater than two standard deviations for every feature extraction approach and breathing pattern. It can clearly be seen that the respiratory belts have issues detecting apnea segments correctly. Breathing patterns with either low RRs or apneic phases yield more outliers. The chest IRT signals yield the least RR extraction outliers compared to the other approaches.

Table 4 compares the RMSE regarding respiratory amplitude extraction for the different modalities and breathing patterns. Here, the chest belt signals are the most accurate. However, border IRT signals also produce excellent results. Only breathing patterns with significant respiratory efforts (hyperpnea and Kussmaul breathing) yield large errors. The tracked marker signals (chest IRT and RGB) also show good accuracy. However, these signals yield significantly larger errors in the case of bradypnea.

### 3.3. Dataset Analysis

**Figure 12:**
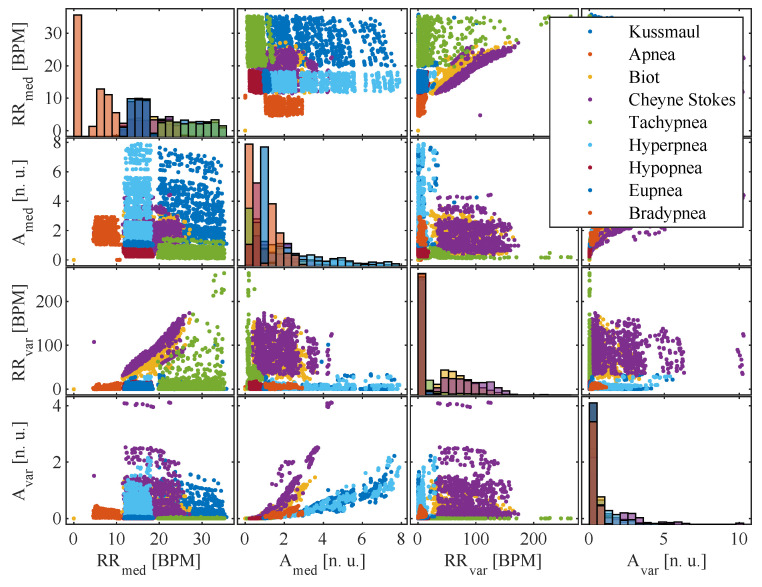
Scatter plots for all extracted respiratory feature combinations.

Figure 12 shows a plot matrix with the scatter plots of all extracted features utilizing their median value over a complete breathing pattern segment. Generally, no distinct lines can be observed in the feature distributions of $RR_{\mathrm{med}}$ and the signal amplitudes $A_{\mathrm{med}}$. Instead, they are evenly distributed over the entire defined range of values. This distribution confirms the effectiveness of the chosen data augmentation principles and prevents the overfitting of classifiers at systematic gaps that would otherwise have arisen in the feature value ranges due to the study design.

The histograms on the main diagonal of the plot matrix show the distribution of all classes along their complete value range. The RR is the feature that can provide the best separation between the displayed pattern classes. However, it is only sufficient for classifying apnea, bradypnea, eupnea, and tachypnea. All other classes need multiple features for their extraction. Here, the most important pair of features appear to be the median RR $RR_{\mathrm{med}}$ and signal amplitude $A_{\mathrm{med}}$, which imply the RE. By adding this feature, hypopnea, hyperpnea and Kussmaul breathing become separable. For complex respiratory patterns, like Biot’s and Cheyne–Stokes breathing, which cover a wide range of RRs and REs, some separation between the other patterns can be established by introducing the variance features.

### 3.4. Classifcation Results

The classification accuracies for the different extraction algorithms and modalities are shown in Table 5. With almost 96 % accuracy, the chest marker tracking approach in IRT videos outperformed the other approaches. Interestingly, all contactless approaches outperformed the gold standard, although it showed equal or better performance during the feature extraction.

**Figure 13:**
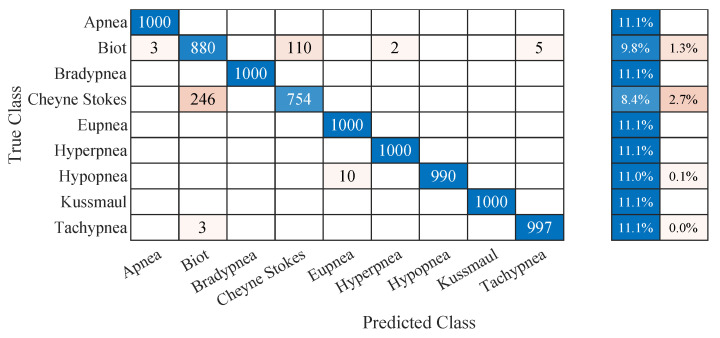
Confusion matrix for the one vs. one multiclass support vector machine breathing pattern classification of the respiratory signal extracted by chest feature tracking in thermal videos.

Figure 13 shows the confusion matrix of the chest IRT dataset. It can be seen that the results are almost perfect. The most significant source of confusion originates from the complex respiratory patterns (Biot’s breathing and Cheyne–Stokes respiration). With confusion rates of 1.3 % and 2.7 %, these breathing patterns were more than 13 times more likely to be misclassified than hypopnea, the next most prominent source of confusion with 0.1 % misclassifications.

## 4. Discussion

The results clearly show the feasibility of classifying and monitoring breathing patterns by using IRT and RGB video recordings. However, it should still be discussed which of the presented approaches is the most promising for timely product development, where the performance differences of the different approaches arise from, and which improvements, respectively, extensions in the field of automated respiration analysis would be conceivable in the future.

During the evaluation, interesting differences regarding the quality of respiratory amplitude or RE extraction became apparent. In contrast to the other modalities and measurement locations, the nose IRT signal could not show any differences in RE over the different breathing patterns. The reason is that the temperature of the inhaled and exhaled air, which leads to temperature changes in the IRT recordings, does not vary with the RE but with the respiratory flow. Although it renders these signals useless for the presented classification approach, it makes them interesting for extended respiratory assessment approaches. For example, monitoring the relationship between respiratory flow and effort yields information about a lung’s mechanical condition regarding resistance and compliance. These two measures are vitally important when diagnosing obstructive or restrictive lung diseases.

The signals from the border RoI approach were inferior compared to the chest-tracker-based approaches. Because all of the extracted raw signals are located on the subject’s chest, they contain more relevant information, compared to the other approach. The border plRoI only deliver signals with relevant respiratory content around the shoulder regions. This fact also explains the observed outliers for the respiratory amplitude extraction: although the chest-tracker-based approaches mainly evaluate chest rise, the border RoI approach focuses on shoulder movement. Healthy subjects increase their lung volume during phases of bradypnea (e.g., during meditation) to maintain a sufficient minute ventilation. The result is a more pronounced chest excursion and, thus, larger signal amplitudes for the chest-tracker-based approaches. On the other hand, the border RoI approach is highly sensitive to the utilization of auxiliary respiratory muscles, resulting in an additional shoulder lift. Hence, the border RoI signals had a significantly higher amplitude during phases with high RE. Again, these differences could be used to improve breathing pattern classification.

There were also notable differences for the measurement modalities themselves: In general, IRT has proven superior to the other modalities as it provides high contrasts. High contrast could not only be leveraged in the border RoI approach, where the motion of the warm subjects in front of a colder background was analyzed, but also for the marker-based approach. Here, cloth wrinkles on the subjects’ chests also created large temperature gradients (see Figure 6), which were beneficial for the tracking algorithms. Although RGB recordings have a higher resolution, the low contrast on single-colored textiles reduces the quality of the feature point tracking algorithm. Furthermore, over- and underexposure can further limit the usable contrast. However, there may exist scenarios where IRT has less contrast than RGB sensors (e.g., outside during hot or cold weather, when either the background has a similar temperature as the subject or the subject wears insulated clothing). Because of the low cost of RGB sensors, it is proposed to use both modalities simultaneously and perform signal fusion to enhance the robustness further. Chest belt sensors have been shown to deliver high-quality signals. However, during apnea, they were prone to artifacts due to baseline wander. In contrast to the other modalities, belt sensors do not feature redundant raw signals, which can be used for implicit detrending through the PCA fusion algorithm. This fact contributed to the inferior performance during phases of apnea.

For breathing pattern classification, the contactless approaches outperformed the respiratory belts, although the chest belt signal showed better performance for RR and RE extraction. The observation that respiratory belt signals showed inferior performance during phases of apnea can not alone explain this fact. Only 0.1 % of false classifications were observed during apnea. The primary error sources were the complex breathing patterns (Biots’s breathing and Cheyne–Stokes respiration). Here, the contactless approaches performed better. A possible explanation might be that the contactless approaches tended to exaggerate high REs, which might be beneficial for classification. Nevertheless, because complex respiratory patterns consist of multiple simple patterns, one-shot classifiers, like the one presented in this manuscript, are not ideally suited for their classification. By using more advanced classifiers, chances are high that all discussed modalities yield better, more comparable results.

Multiple approaches could be tested to improve the classification of complex breathing patterns. The first possibility is the utilization of CWT spectrograms as inputs for a suitable 2D convolutional neural network. This approach would provide the classifier with all relevant time-frequency and amplitude information necessary for successful classification. In addition, the temporal feature signals could be used as input for a suitable recurrent neural network. The memory effect of these neural networks makes them ideal for classifying temporal patterns. The last proposed possibility would be to use the classifier presented here only for the simple breathing patterns and to rely on a simple, downstream recurrent neural network to detect and classify more complex breathing patterns based on its outputs.

## 5. Conclusions

Knowing a patient’s current respiratory pattern can help detect a broad range of medical conditions. Therefore, the relevance of a system capable of continuously classifying a patient’s respiratory pattern unobtrusively and cost-effectively should not be underestimated. This work presented several approaches to extract and classify respiratory signals from RGB and IRT videos. Their performances were compared to respiratory belt sensors located at the chest and abdomen, serving as gold standards. With the chest IRT approach, 95.79 % classification accuracy was achieved. The other contactless approaches, border RoI and chest RGB, performed comparably well and outperformed the gold standard. These promising results clearly show the feasibility of contactless systems for performing a complete respiratory pattern assessment.

Improvements are needed for classifying complex breathing patterns. The dataset created for this study was published to enable the development of other classification approaches and their objective comparison. Expanding the data set to include other breathing patterns, for example, occurring in obstructive or restrictive lung diseases, is a promising prospect for subsequent work, which can further increase the medical usability of the classification results. In a patient study in the recovery room of the RWTH Aachen University Hospital, the results of the presented subject study are currently being compared and validated with actual pathological breathing patterns. The results shall ensure that the dataset and the developed classifiers are suitable for practical use.

## Figures and Tables

**Table 1:** Pattern description of the breath-protocol, shown in Figure 2.

No.	Pattern	Resp. Rate (RR) Breaths per Minute	Resp. Effort (RE) Norm. Units Units	RR Range Breaths per Minute
1	Bradypnea	5	1	5–10
2	Eupnea	12	1	12–18
3	Hypopnea	12	0.25	12–18
4	Hyperpnea	10	2.5	12–18
5	Tachypnea	35	1	20–35
6	Eupnea	15	1	12–25
7	Kussmaul breathing	30	2.5	20–35
8	Cheyne-Stokes respiration	0, 20	0–4.5	12–25
9	Tachypnea	35	0.25	20–35
10	Biot’s breathing	0, 15	1	12–25
11	Biot’s breathing	0, 10	2.5	12–25
12	Apnea	0	0	0

**Table 2:** Root-mean-square errors (RMSE) for the respiratory rate (RR) extraction of the different recorded breathing patterns and modalities. The unit of all values is in Breaths/min.

Pattern	Chest IRT	Border IRT	RGB	Abd. Belt	Chest Belt
Bradypnea	0.33	0.44	0.34	0.42	0.12
Eupnea	0.21	0.21	0.20	0.19	0.05
Hypopnea	0.28	0.36	0.28	0.24	0.10
Hyperpnea	0.21	0.14	0.13	0.24	0.05
Tachypnea	0.24	0.24	0.24	0.88	0.11
Kussmaul	0.40	0.28	0.29	0.37	0.14
Cheyne Stokes	0.56	1.61	0.86	0.88	0.23
Biot’s	1.48	0.52	0.97	3.76	1.59
Apnea	0.00	0.60	1.20	7.43	6.20
Mean	0.41	0.49	0.50	1.60	0.95
Median	0.31	0.40	0.32	0.65	0.13

**Table 3:** Outliers with errors greater than two standard deviations from the mean value for the respiratory rate (RR) extraction of the different recorded breathing patterns and modalities.

Pattern	Chest IRT	Border IRT	RGB	Abd. Belt	Chest Belt
Bradypnea	5	9	7	9	0
Eupnea	1	0	1	2	0
Hypopnea	1	3	3	0	0
Hyperpnea	0	0	0	1	0
Tachypnea	0	0	0	1	0
Kussmaul	0	0	0	0	0
Cheyne Stokes	2	10	2	3	0
Biot’s	7	2	7	29	9
Apnea	0	9	13	210	209
Sum	16	33	33	255	218
Ratio	0.18%	0.37%	0.37%	2.83%	2.42%

**Table 4:** Root-mean-square errors (RMSE) for the respiratory amplitude extraction of the different recorded breathing patterns across all modalities. The unit of all values is in normalized units.

Pattern	Chest IRT	Border IRT	RGB	Abd. Belt	Chest Belt
Bradypnea	0.81	0.26	0.79	0.42	0.34
Eupnea	0.06	0.08	0.09	0.07	0.06
Hypopnea	0.29	0.27	0.36	0.37	0.35
Hyperpnea	0.73	2.57	1.86	0.94	0.77
Tachypnea	0.34	0.29	0.33	0.41	0.29
Kussmaul	0.64	2.26	1.66	1.06	0.83
Cheyne Stokes	0.84	0.85	0.95	1.14	0.90
Biot’s	0.83	0.54	0.74	0.89	0.82
Apnea	0.00	0.01	0.00	0.02	0.01
Mean	0.50	0.79	0.75	0.59	0.49
Median	0.57	0.42	0.75	0.51	0.42

**Table 5:** Breathing pattern classification results for all extracted signals using a one vs. one multiclass support vector machine.

	Chest IRT	Border RoI	Chest RGB	Abd. Belt	Chest Belt
Accuracy	95.79%	94.93%	93.48%	91.87%	92.37%

## Data Availability

The data presented in this study are under submission at PhysioNet.

## Abbreviations

The following abbreviations are used in this manuscript:

PCA	Principal component analysis
KLT	Kanade-Lucas-Tomasi
SVD	Singular value decomposition
RoI	Region of Interest
FFT	Fast Fourier Transform
CWT	Continous Wavelet Transform
RR	Respiratory Rate
RMSE	Root Mean Square Error
WFDB	Waveform Database
RE	Respiratory Effort
IRT	Infrared Thermography
NIR	Near Infrared
SVM	Support Vector Machine

## Appendix A. Experimental Setup

**Table A1:** Devices used for data caption.

Device	Product Name	Manufacturer	Information
Chest Sensor	PVDF Effort Sensor	SleepSense	
Adbodem Sensor	PVDF Effort Sensor	SleepSense	
A/D Converter	NI USB-6008	Nordic Instruments	resolution: 12 bit, sampling rate: 5 kHz
Ring Light	SL-480	Dörr	color temperature: 4600 K, illumination power: 1000 lx
RGB-Camera	Mako G234C	Allied Vision	resolution: 1920 × 1080 px, color depth: 8 bit, frame rate: 15 FPS lens: Kowa LM50HC
Thermal camera	Boson 640	FLIR	resolution: 640 × 512 px, color depth: 16 bit, frame rate: 30 FPS, lens: 12 ${}^{\circ }C$ FOV spectral range: 7,5–13,5 μm

## Appendix B. Used Parameters

**Table A2:** Parameters for Savitzky Golay pre-processing filter.

Order	Frame Length
2	1.5 s

**Table A3:** Parameters for the Peak detection algorithm.

Prominence	Min. Distance	Max. Peak Width
0.3 n.u.	$\frac{60s/\min}{40\mathrm{Breaths}/\min}=1.5s$	$\frac{2}{3}\frac{60s/\min}{4\mathrm{Breaths}/\min}=10s$

**Table A4:** Parameters for the CWT.

Wavelet	Time-Bandwidth Product	Voices per Octave	Frequency range
Morse	10	48	[440]Breaths/min=[0.0670.667]Hz
